# Incidence, Spatial Pattern and Temporal Progress of Fusarium Wilt of Bananas

**DOI:** 10.3390/jof7080646

**Published:** 2021-08-08

**Authors:** Daniel W. Heck, Miguel Dita, Emerson M. Del Ponte, Eduardo S. G. Mizubuti

**Affiliations:** 1 Departmento de Fitopatologia, Universidade Federal de Viçosa, Viçosa 36570-900, Brazil; dwinterheck@gmail.com (D.W.H.); delponte@ufv.br (E.M.D.P.); 2 Bioversity International, Cali 763537, Colombia; m.dita@cgiar.org

**Keywords:** *Fusarium oxysporum* f. sp. *cubense*, Panama disease, epidemiology, disease impact, loss, yield, management

## Abstract

The effective management of Fusarium wilt of bananas (FWB) depends on the knowledge of the disease dynamics in time and space. The objectives of this work were: to estimate disease intensity and impact, and to investigate the spatial and temporal dynamics of FWB. Fields planted with Silk (*n* = 10), Pome (*n* = 17), or Cavendish (*n* = 3) banana subgroups were surveyed in Brazil, totaling 95 ha. In each field, all plants were visually assessed, and diseased plants were georeferenced. The incidence of FWB and the impact of the disease on the yield on a regional scale were estimated. Spatial patterns were analyzed using quadrat- and distance-based methods. FWB incidence ranged from 0.09% to 41.42%, being higher in Silk fields (median = 14.26%). Impacts of epidemics on yield ranged from 18.4 to 8192.5 kg ha^−1^ year^−1^, with an average of 1856.7 kg ha^−1^ year^−1^. The higher economic impact of the disease was observed on Silk cultivar with an average loss of USD 1974.2 ha^−1^ year^−1^. Overall, estimated losses increased on average by USD 109.8 ha^−1^ year^−1^ at each 1% of incidence. Aggregation of FWB was detected by all analytical methods in 13 fields (1 of Cavendish, 11 of Pome, and 1 of Silk). In the other 17 fields, at least one analytical method did not reject the null hypothesis of randomness. One field (5 ha), composed of six plots, was selected for spatial and temporal studies during two years with bi-monthly assessments. A sigmoidal curve represented the FWB progress and the Gompertz model best-fitted disease progress. The level of aggregation varied over time, and evidence of secondary infection to neighboring and distant plants was detected. FWB is a widespread problem in Brazil and yield losses can be of high magnitude. Epidemiology-based management strategies can now be better established.

## 1. Introduction

Banana plants with symptoms of Fusarium wilt were first reported in Australia in 1874 [1]. In 1890, wilted plants were investigated in Cuba where Erwin F. Smith reported the soil-borne fungus *Fusarium oxysporum* associated with symptomatic banana plants [2]. It has been suggested that more than one species of *Fusarium* may be involved with the Fusarium wilt of banana (FWB), also known as Panama disease. *Fusarium oxysporum* f. sp. *cubense* (E. F. Smith) W. C. Snyder and H. N. Hansen has been traditionally reported as the causal agent of FWB, but another species, *F. odoratissimum* N. Maryani, L. Lombard, Kema & Crous, was nominated to describe one of the lineages of *F. oxysporum* capable of causing the disease [3,4,5]. The taxonomy of *F. odoratissimum* is under debate [6] and we prefer to use the traditional and most widely accepted nomenclature for the causal agent of FWB: *F. oxysporum* f. sp. *cubense* (*Foc*).

Fusarium wilt is widespread in all banana-growing areas. The movement of infected planting material was responsible for the fast spread of the disease worldwide [7]. This practice is still common among banana farmers, mainly, but not exclusively, in subsistence systems. Despite the long history of damage by FWB on banana crops, there are important knowledge gaps about basic epidemiological features of FWB, such as intensity, impact, and the spatio-temporal dynamics of epidemics.

Disease spatial pattern is primarily determined by biological and ecological factors correlated to the pathogen life-cycle [8], especially those related to the distribution of the primary inoculum [9]. Knowledge gained from the analysis of spatial patterns may help generate sound scientific hypotheses about pathogen dispersal mechanisms [10,11], and then can support mitigation strategies in the case of introduction of new variants of a pathogen into an area. Additionally, spatial analyses are useful for several purposes including the study of pathogen population dynamics [12], design of experiments [13], sampling programs for disease or pathogen monitoring [14,15,16], assessment of crop losses about disease intensity [13], and the development of management strategies [9].

Disease patterns can be the realization of the dispersal of propagules [17] and despite the epidemiological implications of the understanding of spatial dynamics to disease management, to date, only three studies provided information on the spatial pattern of FWB [15,18,19]. The aggregated pattern of FWB was detected in six banana plots (1334 m^2^ each) assessed in China. The higher the disease intensity, the higher was the aggregation level [15]. FWB was also reported to have an aggregated pattern in banana fields in Australia, but random diseased plants could also be found in some fields [18]. Weevil borers were allegedly involved in the spread of FWB [18]. In Brazil, weevil borers were detected affecting the spatial pattern of FWB [19]. The disease was more aggregated in a field where the population of weevil borers was managed and kept at lower numbers than in the unmanaged field [19]. Another important biological fact is the potential aerial dispersal of the pathogen [20]. If secondary infections could occur from airborne inoculum, a lower degree of aggregation and a higher number of diseased plants scattered in the field would be expected.

Other forms of spore dispersal cannot be ruled out, such as transport by animals, water, soils, substrates, and anthropogenic factors [21]. Cultural practices, such as desuckering (destroying unwanted suckers which develop from the corm of a banana plant) and fertilization of asymptomatic but infected plants and sharing contaminated planting materials and tools, are common practices in banana plantations and contribute to the spread of FWB within and between fields. The movement of symptomless seedlings, infected fruit crowns, leaf trash through shipments, or infested soil adhered to any object used by workers may have facilitated the introduction of *Foc* tropical race 4 (TR4) from Southeast Asia to Africa or the Middle East [22] and even to South America. Spatial analysis may help elucidate whether an observed pattern emerged only by chance or due to an underlying process that may or may not be known. To succeed in such endeavor, it is necessary to use analytical methods based on different approaches for inspecting spatial patterns at different scales since the impact of a pattern on a process can vary with the scale [23].

Classic and current texts emphasize the potential large contribution of infected plants or contaminated tools to the spread of FWB in fields [21,24]. By examining the pattern of FWB epidemics in fields it is possible to infer that autochthonous inoculum of *Foc* would most likely result in random or regular distribution of wilted banana plants, whereas external inoculum from contaminated transplants or tools would result in aggregation or clusters of diseased plants. Pattern analysis and the study of spatio-temporal dynamics of epidemics can shed light on the contribution of the origin of the inoculum of FWB epidemics in banana plantations. However, these studies are largely absent for the *Foc*-banana pathosystem. The objectives of this study can be summarized by the following research questions: (i) What is the intensity and (ii) the impact of FWB in different production regions and cultivars in Brazil? (iii) What is the predominant spatial pattern of FWB epidemics at different scales? (iv) What is the temporal dynamic? Lastly, (v) how does the spatial distribution change over time?

## 2. Materials and Methods

### 2.1. Fields

**Figure 1:**
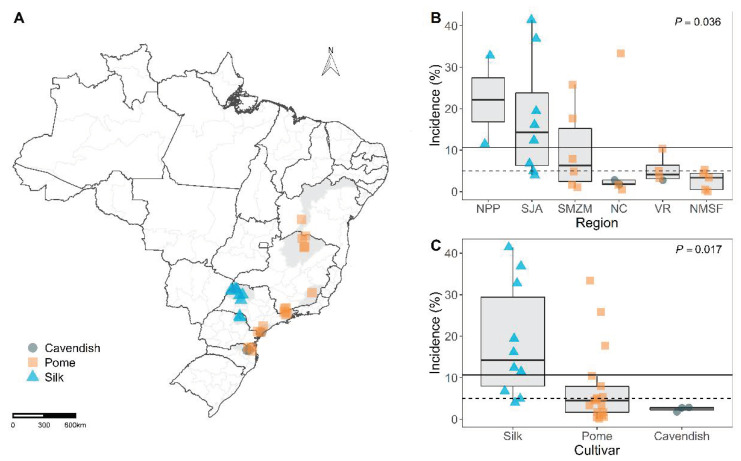
(**A**) Location of the 30 sampled fields distributed in five states of Brazil. Mesoregions are shaded in gray, and the exact location of the fields was identified by geometrical shapes that correspond to banana cultivars. Fields located close to each other appear superimposed on the map and geometric shapes are darker. (**B**) Boxplots illustrate the incidence of Fusarium wilt on banana fields in different regions of Brazil and (**C**) for different cultivars. The average and median values are represented by dashed and solid lines, respectively.

Thirty banana fields with records of FWB incidence were assessed from March 2016 to April 2017. The fields were grouped based on geographic location: Vale do Ribeira (VR; N = 4 fields); São José do Rio Preto and Araçatuba (SJA; N = 8), in São Paulo state; Serra da Mantiqueira and Zona da Mata (SMZM; N = 6), in São Paulo and Minas Gerais states, respectively; Norte de Minas and Vale do São Francisco da Bahia (NMSF; N = 5), in Minas Gerais and Bahia states, respectively; Norte Catarinense (NC; N = 5), in Santa Catarina state; and Norte Pioneiro Paranaense (NPP; N = 2), in Paraná state (Figure 1A). The fields were located within 13°14′ S to 26°28′ S latitude and 43°21′ W to 50°50′ W longitude. Altitude ranged from 45 masl in VR to 1150 masl in SMZM. These five states accounted for 43% of the planted area and 53% of the total Brazilian banana production in 2017 [25]. Field size ranged from 0.85 to 6.67 ha and was planted with Silk (N = 10 fields; a total of 34.7 ha), Pome (N = 17; 51.4 ha), or Cavendish (N = 3; 8.5 ha) banana subgroups.

### 2.2. Disease Intensity

In each field, all banana plants were visually assessed for typical external and internal symptoms of FWB. External symptoms corresponded to plant wilting, yellowing of older leaves, the collapse of leaves at the base of the petiole, fallen and dried leaves around the pseudostem, wrinkling and distortion of leaf blades, and splitting at the base of pseudostem. Internal symptoms were observed when external symptoms were not evident, but the plant appeared infected. A small cut in the pseudostem was made to verify yellow, reddish-brown, or black discolorations in vascular tissues [26]. If external and internal symptoms were present in at least one plant of the mat, the whole mat was considered diseased and was georeferenced using a handheld GPS device (GPSMAP^®^ 64, Garmin, Olathe, KS, USA). When symptoms were not clearly assigned to FWB, samples were collected for further confirmation using morphological and molecular analyses (Heck et al., in preparation). For each field, data sets containing the polygon used to delimit the field perimeter and the diseased plants’ geographic coordinates were used for spatial analyses.

The number of diseased plants (*y*) in each field was recorded, and the estimated incidence ($\hat{p}$) was calculated as $\hat{p}=$ y/$\hat{n}$_i_, where $\hat{n}$ is the estimated number of plants. The $\hat{n}$*_i_* was calculated using the polygon representing the field perimeter (used to estimate the area) and plant spacing (plant distance within and between rows). Disease intensity was compared among regions and cultivars. The data were submitted to Shapiro–Wilk and Bartlett’s tests and transformed by log(*x*) before analysis of variance (ANOVA). Multiple comparison was performed by Tukey HSD test with the *stats* package in the R Software Version 3.6.1 [27]. All the following spatial and temporal statistics were calculated using disease incidence as proportion and in the same version of the R Software. The incidence values were presented as the percentage of infected plants.

### 2.3. Estimated Losses

Yield loss (*L*) per unit area (ha) for each field was calculated based on *L* = *W* ∗ $\hat{p}$, where *W* is the actual yield of the banana crop per year (ton ha^−1^ year^−1^) and $\hat{p}$ is the estimated incidence (proportion) [17]. The actual yield is the average yield of the municipality in which a field was located (Table S1) [25]. Economic loss was calculated as *L* (US$) = *L* ∗ $\bar{P}$ *_s_*, where $\bar{P}$ is the average price for each banana subgroup (*s*). Monthly prices from 2013 to 2016 for each banana variety were obtained from Fala.BR (https://www.gov.br/acessoainformacao/, accessed on 17 March 2021). Linear regression was used to analyze the relationships between yield loss and the disease incidence, *L* = *β*_0_ + *β*_1_ ∗ $\hat{p}$, where *β*_0_ and *β*_1_ are regression parameters [17]. Yield losses were also compared among regions and banana subgroups. The data were analyzed as in Section 2.2.

### 2.4. Spatial Pattern Analyses

Analytical methods based on quadrats and distances were used to investigate the spatial patterns at different spatial and temporal scales.

#### 2.4.1. Quadrat-Based Methods

Incidence maps were transformed into quadrats using the *quadratcount* function of the *spatstat* package in R software [28]. The exact number of plants in each quadrat could not be computed because the rows were not regularly spaced or straightly set in most fields. The maximum number of plants in a quadrat was assumed to vary from 4 plants in 2 × 2 to 36 plants in 6 × 6 quadrat-sizes. The sampling unit area varied among fields because the different spacing between plants was observed, but the relationship of the distance within and between rows was the same. The number of diseased plants in each quadrat was determined and used in the spatial analysis.

*Spatial hierarchy*. Initially, sampling units were set at four hierarchical levels: 2 × 2 (lowest), 3 × 3, 4 × 4, and 6 × 6 (highest) estimated plants per quadrat. Analyses of *β*-binomial curves were used to assess aggregation within-sampling-unit. The spatial hierarchy analysis was performed using *spatial_hier* from *epiphy* package [29].

*Dispersion index*. The index of dispersion (*D*) for binomial data was calculated for each field as the ratio of the observed and the estimated variances [13]. A *χ*^2^ test was performed to test if the dispersion index equals 1 (*D* = 1). The analysis was conducted using the *agg_index* function from the *epiphy* package.

*Fitting distributions*. The binomial and *β*-binomial distributions were fitted to the disease incidence data for each field. The χ^2^ goodness-of-fit test for both distributions and a log-likelihood ratio test (*LRS*) was used to determine whether the *β*-binomial better fits the observed frequency than the binomial distribution. This analysis was performed using the *fit_two_distr* function from the *epiphy* package.

*Binary power law*. The binary power law was used to evaluate the relationship between the observed variance and the corresponding variance on the assumption of a binomial distribution of FWB incidence using an intermediate quadrat-size (3 × 3) [17]. The categorical values referring to the different banana subgroups—Cavendish, Pome, or Silk—were included in data sets. A modified t-test compared the model’s parameter estimates against the null hypothesis and between them [30].

#### 2.4.2. Distance-Based Methods

*Spatial Analysis by Distance IndicEs* (*SADIE*). This method uses the location of the sampling units (quadrats) and the number of diseased individuals inside the unit to analyze the spatial arrangement of the diseased individuals by the distance to regularity (*D_r_*) [31]. The modified index developed by Li et al. [32] was computed by the *sadie* function from *epiphy* package.

*Events and intervals*. The distance-based statistic [17] was used as an alternative to quadrat-based methods. In this analysis, patterns of points were described based on intervals in space among events. The goodness-of-fit of the point process model was performed using a *cdf.test* function from *spatstat* package.

*L*(*d*) *function*. Ripley’s *K*(*d*) function is a cumulative distribution function useful to analyze completely mapped spatial point process data [33]. *K*(*d*) considers the entire distribution of distances rather than just the mean of neighbor events [17,33]. *L*(*d*) is a transformed version of *K*(*d*), on which the expected *K* value is equal to distance [34]. The analyses were performed using *Lest*, *envelope*, and *mad.test* functions from *spatstat* package.

*Semivariance*. The semivariance (*γ*) is a measurement of one-half of the variance between values separated by the same distance in a specific direction. Samples separated at distances greater than the range, may be considered spatially independent [17]. The semivariance and the parameters (range, nugget, and sill) were calculated using the *variog* and *variofit* functions from *geoR* [35].

#### 2.4.3. Concordance Analyses

The fields were classified in aggregated or random as result of spatial statistic and tested by Cohen’s kappa for agreement between pairs of statistical methods and Fleiss’s kappa for an overall agreement among all methods used. The analysis was performed using the *kappa2* and *kappam.fleiss* functions from the *irr* package [36].

### 2.5. Temporal Analyses

One Prata (Pome subgroup) banana field with 4.4 ha established in 2012 in Teixeiras, Minas Gerais state (Brazil), was selected to study the disease dynamics in space and time. The area has been cultivated in a low-input system, with minimal cultural practices. Six plots delimited by dirty roads, ranging from 0.3 to 1.0 ha, were set in the field. The number of plants per plot ranged from 171 to 649. Plants were spaced approximately 3 × 5 m, within and between rows, respectively. The plots were assessed from April 2017 to February 2019 every two months, resulting in 12 disease assessments. Disease incidence was assessed as described above.

Hourly records of temperature, relative humidity, and precipitation data were obtained from the closest standard weather station located at 14.4 Km from the field. Data were provided by Instituto Nacional de Meteorologia (INMET) of Ministério da Agricultura, Pecuária e Abastecimento (MAPA).

The cumulative incidence was calculated for each plot in different assessment times. The monomolecular, logistic, and Gompertz models were fitted to the disease incidence and plotted over time by nonlinear regression analysis using the *nlsLM* function from the *minpack.LM* package [37]. The best model was chosen based on the lower root mean square error (RMSE), independence and homogeneity of variances, and higher coefficient of determination (*R*^2^).

### 2.6. Spatio-Temporal Dynamics

Spatio-temporal analyses were performed for the same six plots assessed in Teixeiras for temporal analyses. The index of dispersion, *SADIE*, binary power law, and spatio-temporal association analyses were used to describe the spatial pattern of the disease on the plots over time. A spatio-temporal association study assesses the significance of the correlation coefficient between two spatially autocorrelated processes [38,39]. The measurement of the degree of local clustering in sampling units was used to calculate the similarity in the cluster indices of two sequential assessments. The local clustering (*χ_p_*) was obtained by *SADIE* analysis [32], and the overall association, *X*, was acquired by the correlation coefficient of the local clustering between pairs of assessments. The *t*-test corrected for the spatial association was used to analyse the significance of *χ*^2^ distribution [39]. The analysis was performed using the *sadie* and *modified.ttest* functions from *epiphy* and *SpatialPack* [40] packages, respectively.

## 3. Results

### 3.1. Disease Intensity

FWB incidence ranged from 0.09 to 41.4% across the fields located in different regions in Brazil (Table S1). The mean and median incidence values were 10.7% and 5%, respectively (Figure 1B, Table 1). The incidence varied among regions (*p* = 0.036). The highest average incidence was 22.1% in NPP (*n* = 2 fields) followed by SJA (*n* = 8) with an average of 17.8%. In other regions, the average incidence was lower than the overall average of 10.7% (Figure 1B).

The incidence of FWB varied among cultivars (*p* = 0.017). The highest value of average incidence was observed for Silk (*n* = 10 fields), 18.62%. The widest range of incidence values was recorded on Silk fields: minimum of 4% and a maximum of 41.4%. Pome fields (*n* = 17) had intermediate incidence values: average of 7.5%, and ranging from 0.1% to 33.4%. The lowest disease incidence values were recorded on fields planted with Cavendish (*n* = 3) with an average of 2.4% and ranging from 1.8% to 2.8% (Figure 1C).

### 3.2. Estimated Losses

**Figure 2:**
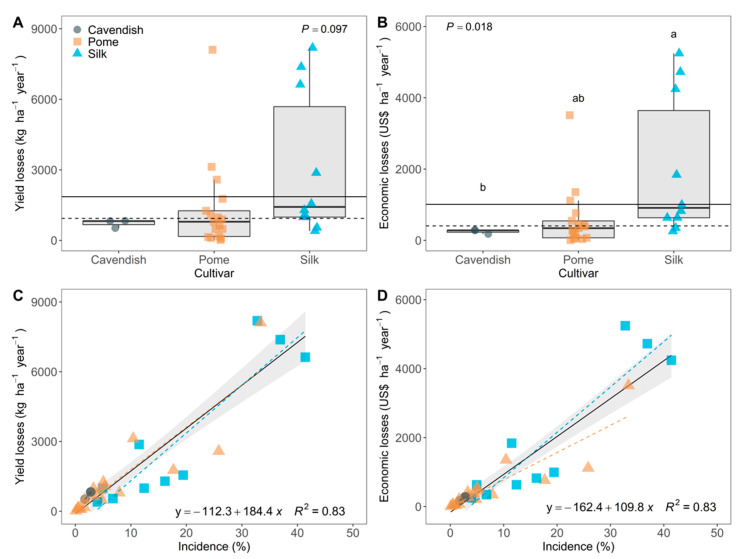
Estimated values of yield and economic losses and its relationship with the incidence of Fusarium wilt of banana assessed in 30 fields from different cultivars and regions of Brazil. (**A)** Frequency of estimated values of yield losses, and (**B**) economic losses by cultivar. Estimated values for each assessed field (symbols) and overall regression (solid black) and cultivar regression lines (dashed colored) for the relationship between FWB incidence and (**C**) yield losses, and (**D**) economic losses. Confidence intervals (CI95%) is presented in (**C**,**D**) as shaded areas.

The yield losses in the 30 fields ranged from 18.4 to 8192.5 kg ha^−1^ year^−1^ (Figure 2A). The mean value was 1856.5 kg ha^−1^ year^−1^ with a median of 935.2 kg ha^−1^ year^−1^. Yield losses did not vary among banana subgroups (*p* = 0.10). Economic losses reached a maximum of USD 5244.8 ha^−1^ year^−1^, with an average value of USD 1010.4 ha^−1^ year^−1^ and a median of USD 405.1 ha^−1^ year^−1^ (Figure 2B). Differences were observed for economic losses (*p* = 0.01) among banana subgroups. Silk differed (*p* = 0.016) from the Pome subgroup with median loss of USD 910.6 ha^−1^ year^−1^ and USD 343.1 ha^−1^ year^−1^, respectively.

A positive linear trend was detected for the relationships between FWB incidence and both the estimated yield and economic losses (Figure 2C,D). The intercept parameter of both regression models, *β_0yield_* = −112.3 (SE = 250.3) and *β_0US$_* = −162.4 (SE = 151.4), did not differ from 0 (*p* > 0.29). The slope parameter of both regression models, *β_1yield_* = 184.4 (SE = 15.8) and *β_1US$_* = 109.8 (SE = 9.53), differed from zero (*p* < 0.001). The 95% confidence interval (CI 95%) of the slope for estimated yield losses ranged from 152.1 to 216.7 kg ha^−1^ year^−1^, and for economic losses from 90.3 to 129.4 USD ha^−1^ year^−1^. The effects of banana cultivars were not significant (*p* > 0.16).

### 3.3. Spatial Pattern Analyses

#### 3.3.1. Quadrat-Based Methods

**Figure 3:**
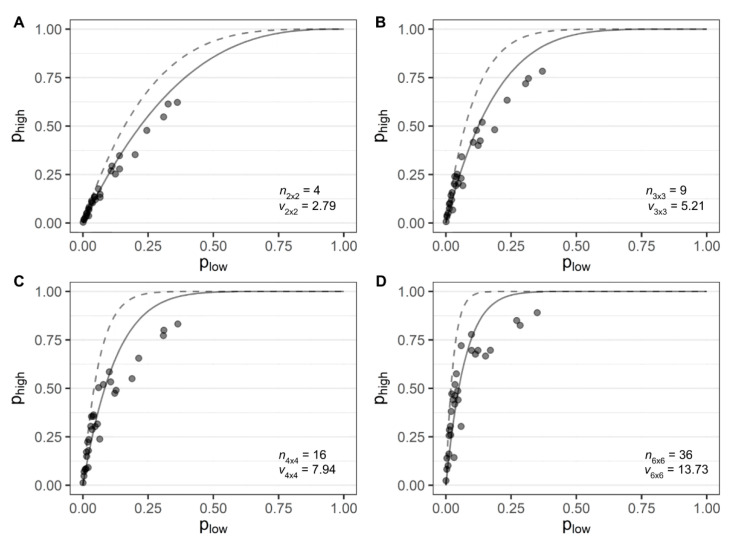
Relationships between the incidence of Fusarium wilt of banana at (**A**) 2 × 2; (**B**) 3 × 3; (**C**) 4 × 4; and (**D**) 6 × 6 quadrat dimensions in 30 banana fields. The sampling unit (quadrat) was the highest (*p*_high_) hierarchical level and the individual (plants) was the lowest (*p*_low_). Binomial (dashed-lines) and *β*-binomial (solid-lines) distributions were fitted to the data. The number of individuals (*n*) and the effective sample size (*v*) estimated at each level are presented in the graphs.

At all four hierarchical levels, the *β*-binomial curves fell under the binomial curves (Figure 3). Values of *v*, interpreted as the effective sample size, were lower than the real number of individuals (*n*) for all hierarchical levels (*p* < 0.001). Effective sample sizes were estimated at *v*_2×2_ = 2.79 (± 0.12) for the 2 × 2 quadrat-size containing 4 individuals, *v*_3×3_ = 5.22 (±0.32) for quadrats of 9 individuals, *v*_4×4_ = 7.95 (± 0.67) for quadrats of 16 individuals, and *v*_6×6_ = 13.73 (± 1.48) for the 6 × 6 quadrat-size containing 36 individuals at the highest level (Figure 3). These values of v correspond to 69.8%, 57.9%, 49.7%, and 38.2% of n in the four hierarchical levels, respectively. Only the intermediate quadrat-size (3 × 3) was used in subsequent quadrat-based analyses.

**Figure 4:**
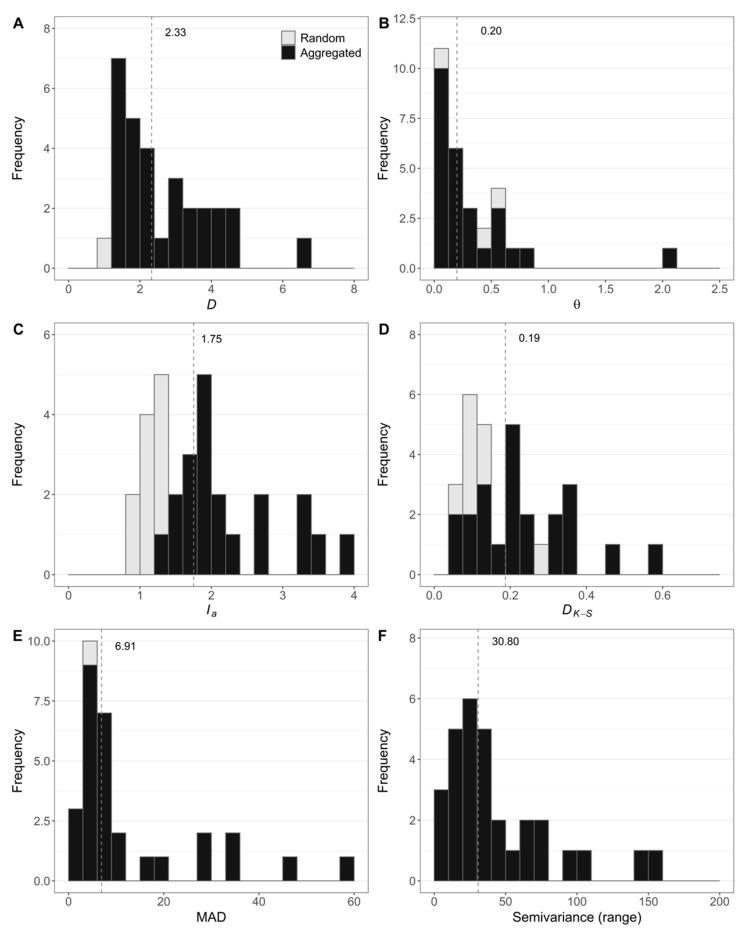
Histograms of (**A**) the index of dispersion (*D*); (**B**) *β*-binomial parameter (θ) from distribution fitting; (**C**) aggregation index (*I_a_*) from Spatial Analysis by Distance IndicEs (SADIE); (**D**) events and intervals (*D_K-S_*); (**E**) Maximum Absolute Deviation (MAD) test of *L*(*d*) function; and (**F**) range from semivariance analyses of Fusarium wilt of banana. The frequencies were based on 30 fields assessed for incidence of Fusarium wilt of banana in Brazil. Median value of the corresponding statistic is presented numerically on the graphs (dashed line). The classification of fields is represented as random (gray) or aggregated (black).

The dispersion index (*D*) ranged from 1 to 6.5 and the median value was 2.3 (Figure 4A). Overall, there were no differences among regions regarding *D* (*p* = 0.119) and aggregation was inferred in all fields and regions, but for a single field (out of 5) in the NMSF region where a random pattern of FWB was detected (Table 1).

The frequency of diseased plants in quadrats was well described for 90% of the fields by the *β*-binomial distribution (*p* > 0.05) (Table 1). For one field the frequency was better described by the binomial distribution (data not shown). The *θ* parameter of the *β*-binomial distribution ranged from 0.02 to 2.01 (median = 0.20) (Figure 4B). There were no differences among regions for the θ parameter (*p* = 0.26). The distribution of diseased plants was better described by the *β*-binomial than by the binomial distribution in 96.7% of the fields (*p* < 0.05) (Table 1). The only case where the LRS was not significant, i.e., the binomial and *β*-binomial models could both describe the distribution of diseased plants, was in a Pome field located in NMB where the FWB incidence was very low (0.5%).

**Figure 5:**
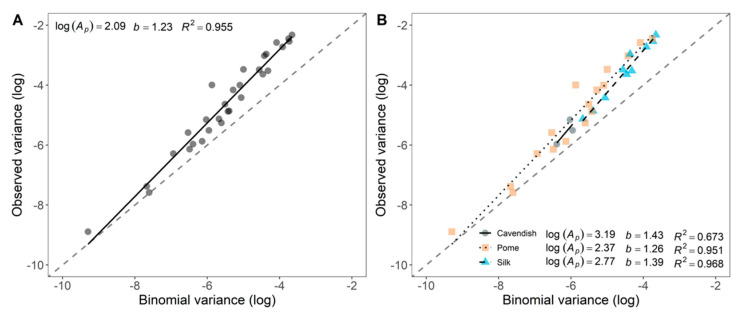
Relationship between the logarithm of the observed variance ($log\left(s_{obs}^{2}\right)$) and the logarithm of theoretical variance ($log\left(s_{bin}^{2}\right)$) for incidence data of Fusarium wilt of banana in 30 fields in Brazil. (**A**) Linear regression for all 30 fields assessed for disease incidence (solid dark line); and (**B**) for Silk (*n* = 10 fields), Cavendish (*n* = 3) or Prata (*n* = 17) cultivars. Binomial lines are represented by dashed gray lines. Binary power law parameters (log(*A_p_*) and *b*) were presented.

The relationship between the logarithm of the observed and theoretical variances for binomial data from 30 fields was well described by the binary power law (*R*^2^ = 0.955) (Figure 5A). The overall estimates of binary power law parameters, log(*A_p_*) (2.09 ± 0.28) and b (1.227 ± 0.05), were significantly (*p* < 0.001) higher than 0 and 1, respectively. The disease had an aggregated pattern and the degree of aggregation was influenced by incidence. FWB had an aggregated pattern in Silk and Pome fields (*p* < 0.001) (Figure 5B). The estimated parameters from the binary power law differed for Silk and Pome (*p* < 0.03). In general, the level of aggregation was higher and more influenced by incidence in Silk (log(*A_p_*) = 2.77 ± 0.41, *b* = 1.40 ± 0.09) than in Pome fields (log(*A_p_*) = 2.37 ± 0.45; *b* = 1.26 ± 0.07). Cavendish fields did not differ from Silk and Pome for both parameters (log(*A_p_*) and *b*; *p* > 0.05).

#### 3.3.2. Distance-Based Methods

The distance to regularity (*D_r_*), computed by SADIE, was calculated for all fields. The aggregation index (*I_a_*) ranged from 0.93 to 3.86 and the median value was 1.75 (Figure 4C). Differences among regions were not observed for *I_a_* (*p* = 0.114). Considering the 30 fields in six regions, the random pattern of FWB was inferred for 10 fields (33.3%) (Table 1). Five of them were located at SJA, all planted with Silk cultivar.

The maximum difference (*D_K-S_*) between the observed and expected distributions ranged from 0.064 to 0.566 (median = 0.187) (Figure 4D). The hypothesis of randomness for Kolmogorov–Smirnov statistic was rejected for 73.3% of the fields (Table 1). The smallest values of *D_K-S_* were observed in SJA and NC (mean of 0.130). In SJA and NC a random pattern of FWB was inferred in almost all fields and there was no evidence to reject the null hypothesis in 50% and 60% of the fields, respectively. In the NMSF region, a random pattern of FWB was inferred in a single field with an intermediate value for *D_K-S_* (0.214) and the lowest FWB incidence (0.09%).

**Figure 6:**
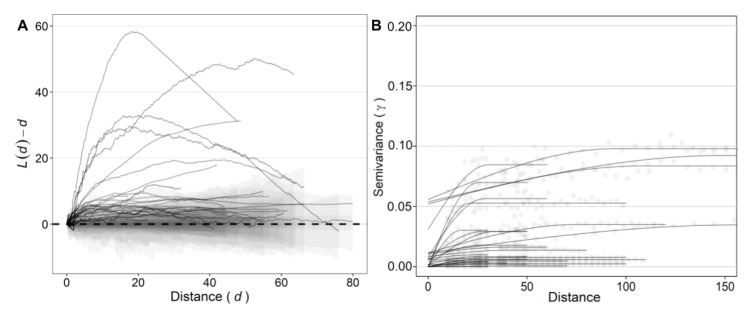
Distance-based statistics of the incidence of Fusarium wilt of banana in 30 fields in Brazil. (**A**) *L*(*d*)-*d* against *d* (solid line), superimposed envelopes (shaded area) and the random pattern (dashed line) are presented. (**B**) Semivariance (*γ*) in a range of distances was represented by spherical model fitted to the data. Fields were represented by solid lines. Distances are presented in meters (m).

The second-order point-pattern analysis used Ripley’s *K* and the linearized form *L*(*d*) function. The maximum absolute deviation (MAD) test ranged from 1.61 to 58.34 m with a median of 6.91 m (Figure 4E). The observed values of *L*(*d*)-*d* for 96.7% of the fields were higher than the critical values of the simulated envelope and resulted in aggregation (Figure 6A). Only one field resulted in a random pattern (*p* = 0.34). This field was cultivated with ‘Pome’ cultivar, had a low incidence of FWB (0.49%), and the random pattern was also inferred by two quadrat-based statistics (*D* and LRS). The distance that resulted in the highest scale of aggregation (max *L*(*d*)-*d*) for each field is shown in Figure 6A.

Semivariance was successfully performed by the spherical model in 27 of 30 fields (Figure 4F). Three fields (10%) had higher spatial variability at scales smaller than the distance between sampling units and the semivariance parameters could not be computed. Semivariance parameters varied among fields. Those with higher incidence had higher values of semivariance (R = 0.87; data not shown). The nugget parameter ranged from 0 to 4.62 with a median of 0.40 and sill ranged from 0.01 to 5.06 (median of 0.46) (Figure 6B). The observed values of the range parameter varied from 9.36 to 154.61 m with a median of 30.80 m (Figure 4F and Figure 5B).

#### 3.3.3. Concordance Analyses

The FWB epidemics had either random or aggregated patterns. Cohen’s Kappa paired agreement was weak or non-significant for most of the spatial statistics (Table 2). Two pairwise tests had a significant agreement. One was a strong agreement (1; *p* < 0.001) between the aggregation index (*D*) and the MAD test on which all fields are classified in the same category (aggregated). The second was a weak agreement of SADIE’s aggregation index (*I_a_*) with *D_K-S_* (0.37; *p* = 0.04), with 22 of 30 fields presenting the same spatial pattern. Fleiss’s Kappa (overall agreement) did not reject the null hypothesis for a random classification of all spatial analyses (*p* = 0.63). The agreement could not be detected by the test in 43.3% (13/30) for the fields that presented the aggregated patterns and none of them were classified as random for all spatial statistics (Table 2).

### 3.4. Temporal Analyses

**Figure 7:**
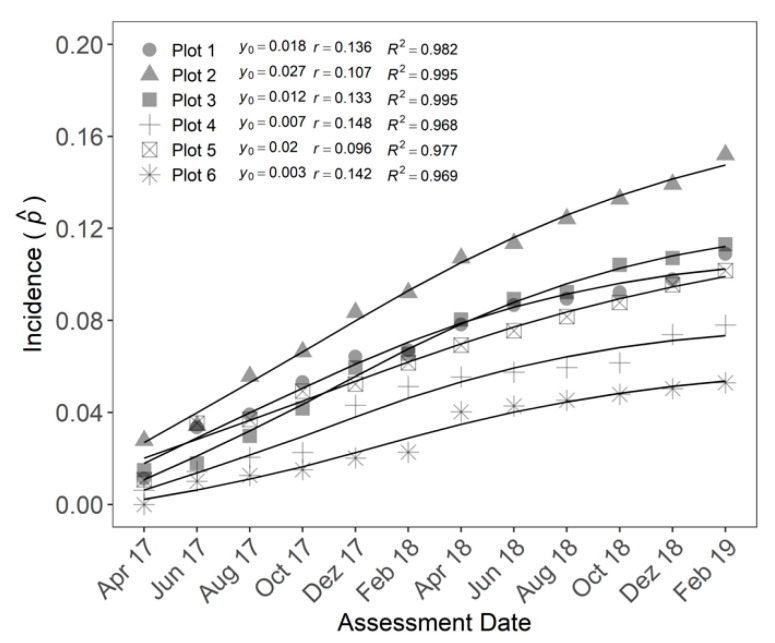
The Gompertz model adjusted to incidence data of Fusarium wilt of banana (FWB) from April 2017 to February 2019 in six plots located in Teixeiras, Minas Gerais, Brazil. Symbols represent the observed incidence in proportion ($\hat{p}$) and solid lines are the predicted incidence of FWB by the Gompertz model.

On the first assessment, the incidence ranged from 0 to 0.03 with a mean and median both of 0.01 (Figure 7). Five of six plots already had symptomatic FWB plants before the beginning of the assessments. After six assessments the incidence in all plots ranged from 0.02 to 0.09 with a median of 0.06 and in the last assessment the FWB incidence ranged from 0.05 to 0.15 with a median of 0.1. The Gompertz model best fitted the disease incidence data over time in all plots (Table S2). The estimated parameters of the model, as the initial incidence (y_0_) ranged from 0.003 to 0.027 with an average of 0.015 and the disease progress rate (r) from 0.096 to 0.148, with an average of 0.127 (Figure 7).

### 3.5. Spatio-Temporal Dynamics

**Figure 8:**
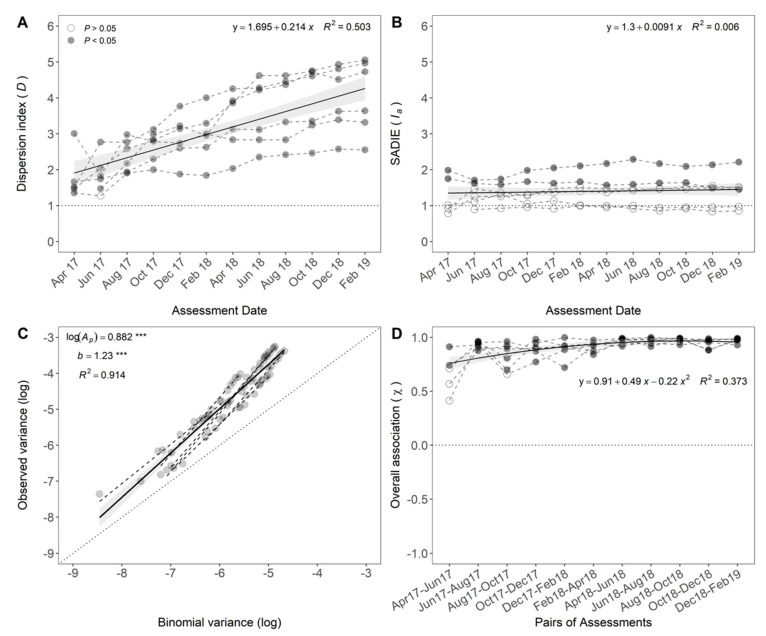
Spatio-temporal statistics were used to characterize the dynamics of Fusarium wilt of banana in six plots assessed in Teixeiras, Minas Gerais, Brazil, from April 2017 to February 2019. (**A**) Dispersion index (*D*); (**B**) aggregation index (*I_a_*) of Spatial Analysis by Distance IndicEs (SADIE); (**C**) binary power law; and (**D**) spatio-temporal association (*X*) of successive assessments of clustering indices. Open and closed dots were presented when the null hypothesis of randomness is not rejected (*p* > 0.05) or rejected (*p* < 0.05), respectively. Average statistic for all plots (solid line) and confidence interval (CI 95%; shaded area) are presented. Equation or summary statistics were presented when applicable. *** Significantly different from zero (log(*A_p_*)) or 1 (*b*) by the modified *t*-test (*p* < 0.001) [30].

Among the different quadrat-based methods, the aggregation index (*D*) was chosen to assess the spatial pattern of FWB at a small scale. In 11 of 12 assessments, banana plants with symptoms of FWB were aggregated in all six plots (Figure 8A). In the first assessment conducted in April 2017, *D* ranged from 1.35 to 3.00 with a median value of 1.52. In the last assessment, February 2019, *D* ranged from 2.55 to 5.06 with a median of 4.18. *D* increased linearly with the FWB progress (*p* < 0.001).

Among the distance-based methods, SADIE was the method used to characterize the heterogeneity of patches and gaps of diseased banana plants over time. The *I_a_* differed from 1 (*p* < 0.05) in only two plots, which correspond to 33.3% of the plots analyzed (Figure 8B). Four plots did not differ from a random pattern (*p* > 0.05). *I_a_* did not change during the period of study (*p* = 0.51). In the first assessment, April 2017, *I_a_* ranged from 0.78 to 1.98 with a median value of 1.01. In February 2019, *I_a_* ranged from 0.86 to 2.21 with a median of 1.47.

The relationship between the logarithm of observed and binomial variances for six plots in bimonthly assessments was well described by the binary power law (*R*^2^ = 0.914) (Figure 8C). Overall, parameter estimates for power law, log(*A_p_*) = 0.882 ± 0.029, and *b* = 1.23 ± 0.045, were significantly different from 0 and 1, respectively, when all plots were jointly analyzed (*p* < 0.001). As log(*A_p_*) was higher than 0 and *b* higher than 1, the pattern of FWB was inferred to be aggregated and the degree of aggregation varied with disease incidence.

Spatio-temporal associations were detected between the pairs of successive assessments for the local clustering (*X_p_*, *p* < 0.05) (Figure 8D). In the first and second assessments, April and June of 2017, three of the five plots had no association (*p* > 0.07). At this time, the overall association of the clustering index (*X*) values ranged from 0.41 to 0.91 with a median of 0.71. After the third association, August 2017 and October 2017, all pairwise comparisons were significantly associated for all plots (*p* < 0.05). In the last association, the *X* values ranged from 0.92 to 0.99 (Figure 8D).

## 4. Discussion

The gap of knowledge related to the spatio-temporal dynamics of FWB and the lack of estimates of the economic impact of the disease have been raised in previous studies [21]. Based on the disease distribution within fields and regions, hypotheses can be developed to understand the ways plant pathogens can be dispersed. The dynamics of the disease over time and information on the social and economic impact of a plant disease are key for implementing resources and efforts to guide strategies to manage plant diseases [41]. Regarding those assumptions, this work assessed the intensity, investigated the spatial and temporal dynamics, and estimated the yield and economic impact of FWB in 30 banana fields (94.6 ha) in six different production regions in Brazil. In total, 109,280 plants were visually assessed, and 7941 symptomatic plants were georeferenced.

The incidence of FWB on the assessed fields in Brazil was moderate (average = 10.7%) when compared to surveys performed in some regions in Africa [42,43]. FWB epidemics reach up to 77% of incidence in Southwest Ethiopia, with an average of 17.8% and a prevalence of 67% in plots (100 m^2^) studied in 180 peasant farms [42]. In East and Central Africa, disease incidence was greater than 40% in most of the fields [43]. Considering disease incidence, this study used a census to characterize the FWB epidemics in the fields. The highest FWB incidence was observed in NPP and SJA regions where there is a higher proportion of fields planted with the highly susceptible cultivar, Silk. Pome and Cavendish fields were significantly less affected by the disease, as these cultivars are moderately and highly resistant, respectively, to the *Foc* populations present in Brazil [21,44]. However, this scenario might change if *Foc* TR4 surpasses the borders with Colombia [45] and Peru [46], or if it is accidentally introduced in Brazil in other ways.

Despite occurring at moderate intensity, FWB may have socio-economic impacts because many affected fields in the study are in low- or moderate-input farms, where the resource access to manage and reduce the disease impact is limited [41]. Overall, at each 1% of FWB incidence detected in the fields resulted in a loss of 184 kg ha^−1^ year^−1^. The average value of ~10.7% of FWB incidence, which results in a yield loss of 1856.5 kg ha^−1^ year^−1^, or USD 1010.4 ha^−1^ year^−1^ can substantially reduce the revenue from the crop. Bananas are cultivated on 468,000 hectares in Brazil [25]. In a hypothetical scenario where 20% of the banana fields in Brazil are affected by FWB with the average incidence observed on this study, the estimated losses would be 173,771.7 tons and USD 94.58 million yearly. Considering that Brazil has one of the highest per capita consumption (~60 kg; FAO: http://www.fao.org/economic/est/est-commodities/bananas/bananafacts/en/#.YOnduejMPDc, accessed on 5 July 2021), the potential losses could feed 2.9 million people yearly. Unfortunately, some crucial information, such as local attainable yield and prices, and either the FWB prevalence in Brazil, were not available for a precise estimation of the social-economic impact of the disease for farmers and consumers. Thus, the estimates may be used as a baseline for farmers, industry, and policymakers. Most likely, the real impact of FWB in the banana industry is still underestimated.

Regarding the spatial distribution of FWB, the aggregated pattern was detected in 43% of the fields by all spatial statistics and the random pattern was detected in 57% of the data sets by one or more analytical methods (Table 2). Some of the quadrat-based statistics (*D* and distribution fitting) resulted in a higher number of fields with an aggregated pattern compared to the distance-based methods (Table 2). The quadrat-based methods do not use the spatial information of the sampling units [31] and have limited capacity to describe the spatial pattern in the entire field. Inferences about heterogeneity are made at scales below the threshold at which the data were collected. However, these tests were important to detect the degree of heterogeneity within disease clusters when few clusters were present. When a quadrat-based method is used in association with distance-based methods the entire process of disease spread could be better studied. Notwithstanding, a clear relationship between quadrat- and distance-based methods was not expected because of the intrinsic features adjusted for the different physical scales set to study spatial heterogeneity [11,47].

Overall, FWB had an aggregated pattern, but clusters of diseased plants (foci) were randomly distributed in the field. Thus, the spatial distribution of the disease is largely driven by the distribution of the initial inoculum in the area. From a focus, neighboring plants are more likely to become infected originating the aggregated pattern. Consequently, a random pattern is likely to be detected at lower intensities, and aggregation may be detected after epidemics develop. These dynamics were revealed when analyzing the data with the binary power law: fields with lower incidences had weaker aggregation parameters than fields with higher incidence values observed in this study (Figure 5A). However, this fact was only observed because the highest incidence was 41%. For foliar pathosystems where disease incidence above 50% is commonly observed, the binary power law’s aggregation parameter is low in datasets with either very low or very high incidences [11,16]. Diseases caused by pathogens dispersed mostly from plant-to-plant usually present this pattern [48,49]. Coffee wilt, caused by *F. xylarioides*, and Fusarium crown and root rot of tomato, caused by *F. oxysporum* f. sp. *lycopersici* (*Fol*), illustrate this pattern of distribution [48,49]. An aggregated pattern of FWB was also found in Australia by the joint-count statistic [18].

The random pattern of FWB epidemics detected in some of the fields by one or more spatial statistics may also warn about other processes affecting the dispersal of *Foc* to sites far from the main foci. Based on the semivariance analysis, half of the fields had a range of values shorter than 30.8 m, meaning that the foci size on these fields are usually smaller than that. The range parameter is the maximum distance that the samples are spatially correlated [50]. The spread of Fusarium crown and root rot in tomatoes ranged from 1.1 to 4.4 m [49]. Tomatoes are an annual crop with a short cycle and fields are normally cultivated at distances less than 50 cm within a row. Bananas are a semi perennial crop cultivated at within row distances that range from 1.5 m to 7 m. In addition to the plant-to-plant spread, weevil borer, other animals, cultural practices, wind, runoff, or irrigation water may contribute to disease dissemination [21]. Aerial dispersal of *Fol* and *F. oxysporum* f. sp. *cucumerinum* has been reported [51,52]. Evidence of external sporulation of *Foc* in banana plants may suggest the potential role of wind and rain dispersion [20]. It is hypothesized that feral pigs surrounding the fields [53] and humans [54] are involved in the within and among field dispersal of *Foc* [7,21]. Insects as weevil borers can carry infective propagules of *Foc* [55] and their populations were driving FWB epidemics in banana fields [19].

Many biological, cultural, and ecological processes can directly affect the spatial pattern of plant diseases [8]. The study of the effect of cultivars in the distribution of FWB indicated that the disease has an aggregated pattern and the level of aggregation varied with incidence. The power law parameter, log(*A_p_*), was significantly different between Silk and Pome cultivars. Silk is more susceptible than Pome, thus a clearer aggregated pattern was more often observed in fields planted to the latter. Based on this pattern, it is possible to infer that FWB can spread farther when epidemics occur in highly susceptible cultivars. Plants with intermediate resistance levels are less affected by FWB up to certain densities of inoculum [21]. In this way, the inoculum density: disease intensity (ID:DI) relationship may hold for FWB as reported in other *F. oxysporum* pathosystems [56,57,58,59].

Soilborne pathogens give rise, in general, to monocyclic diseases [60]. However, multiple cycles of infection may occur in banana plantations affected by *Foc* [7,61]. The sigmoidal pattern observed for the plots of incidence over time suggests that multiple infections may have occurred in the FWB epidemics along the two years of study. The semi-perennial nature of the banana plant and the impractical task of pathogen elimination from the soil means that once infected and infested by *Foc*, respectively, they may act as an inoculum source for an undetermined time. The intensive management to achieve high productivity and other mechanisms of dispersal during the growing season could affect the dynamics of the disease and were reported for other members of *F. oxysporum* [49,52,62]. A similar argument was suggested for *Foc* in banana plantations [18,49,52,62]. The field monitored was managed in a low-input system, so anthropogenic factors, such as cultural practices, were not performed before and during the study period. In this case, *Foc* dispersal is due to natural causes, such as root-to-root contact, vectors, floods, or wind. A critical point of this result is that the highest incidence value observed for the FWB epidemic was low ($\hat{p}$ = 0.15), and the full temporal dynamic of the epidemic (0 to 1) could not be completely characterized. Future studies addressing the temporal dynamic in different cultivars, pathogen populations, abiotic factors, and management practices can complement the epidemiological knowledge of the disease.

The spatio-temporal dynamics support the occurrence of secondary infection as indicated by the spatial and temporal analyses conducted separately. The ratio between the observed and expected variances (*D*) increased over time (Figure 8A). However, the aggregation index (*I_a_*) of a geospatial statistic (*SADIE*) remained unaltered (Figure 8B). These facts showed a low level of aggregation, i.e., low number of diseased plants per sampling unit, at the beginning of the epidemics. Over time, the number of diseased individuals increased on the sampling units where diseased plants were already detected, increasing the ratio between the observed and expected variances, with no or little effect in the geospatial distribution of the foci. The observed pattern agrees with disease transmission to neighboring plants at a higher rate than to plants located farther apart in the field, mainly due to plant-to-plant spread. In addition, temporal associations of the local clustering indices indicated strong evidence of correlations between the successive pairs of assessments. Only in the beginning of the epidemic, when the incidence was low, there was no association in some plots (Figure 8D). However, a lack of association between the first and the intermediate, and the first and the last assessment was observed (data not shown). Mechanisms of *Foc* dispersal to long distances (beyond the borders of the sampling unit) also affected the epidemics and were evidenced in the long term. This fact may be associated with disease spread beyond the borders of sampling units, by the increase in foci size or spread to plants far from initial foci that could not be detected by other methods. Understanding how the inoculum arrives in the field and the mechanisms involved in *Foc* dispersal are key to propose efficient management strategies to reduce the impacts of FWB epidemics.

In summary, the intensity of FWB in Brazil was moderate (average of 10.7%) with losses up to 8.19 ton ha^−1^ year^−1^. The disease distribution was predominantly aggregated with some fields presenting a random distribution of disease foci, which may be due to the way the initial inoculum was introduced and distributed. The polycyclic model best describes the initial development of the epidemic. After establishing the disease in the field, transmission to the neighboring plants seems to drive the epidemics in the short term, while the spatial distribution of foci is mainly affected by long-distance dispersal mechanisms. These insights about the epidemiology of FWB can help banana farmers improve their decisions to manage the disease and reduce crop losses. Assuming the general pattern of non-TR4 populations of *Foc* could be used as a proxy, this large-scale study is also useful to policymakers in charge of the formulation of actions to mitigate the consequences of the introduction of *Foc* TR4.

## Figures and Tables

**Table 1:** Percentage of fields with significant aggregation by the index of dispersion (*D*), *β*-binomial parameter (*θ*), log-likelihood ratio statistic (LRS), index of aggregation (*I_a_*) of the Spatial Analysis by Distance IndicEs procedure (*SADIE*), events and intervals (*D_K-S_*), and linear transformation of Ripley’s *K* function (*L*(*d*)) to each region assessed for incidence of Fusarium wilt of banana in Brazil ^a^.

Region ^b^	*D* ^c^	*θ* ^c^	LRS ^c^	*I_a_* ^c^	*D_K-S_* ^d^	*L*(*d*) Function ^d^
SMZM (*n* = 6)	100	100	100	66.7	100	100
SJA (*n* = 8)	100	75	100	37.5	50	100
NPP (*n* = 2)	100	100	100	50	100	100
VR (*n* = 4)	100	100	100	100	100	100
NMSF (*n* = 5)	80	80	80	80	80	80
NC (*n* = 5)	100	100	100	80	40	100
Overall (*N* = 30)	96.7	90	96.7	66.7	73.3	96.7

^a^ The aggregated pattern was inferred when the null hypothesis of randomness was rejected (*p* < 0.05). The null hypothesis of the *β*-binomial parameter (*θ*) is aggregation (*p* > 0.05). ^b^ SMZM—Serra da Mantiqueira and Zona da Mata; SJA—São José do Rio Preto and Araçatuba; NPP—Norte Pioneiro Paranaense; VR—Vale do Ribeira; NMSF—Norte de Minas and Vale do São Francisco da Bahia; NC—Norte Catarinense. ^c^ Quadrat-based statistics with quadrat-size of 3 × 3 plants. ^d^ Distance-based statistics.

**Table 2:** Cohen’s and Fleiss’s Kappa agreement and proportions of fields with the same classification for the index of aggregation (*D*), *β*-binomial distribution, index of aggregation (*I_a_*) of Spatial Analysis by Distance IndicEs (*SADIE*), events and intervals (*D_K-S_*) and Maximum Absolute Deviation (MAD) test from *L*(*d*) function for 30 fields assessed for Fusarium wilt of banana (FWB) incidence in Brazil ^a^.

Spatial Statistic ^b^	*θ*	*I_a_*	*D_K-S_*	MAD
*D*	−0.05 (26/30)	−0.06 (19/30)	−0.06 (21/30)	1.00 (30/30)
*θ*		−0.18 (17/30)	−0.17 (19/30)	−0.05 (26/30)
*I_a_*			0.37 (22/30)	−0.06 (19/30)
*D_K-S_*				−0.06 (21/30)
Overall ^c^	−0.027 (13/30)			

^a^ The pattern of FWB in the fields was classified as aggregated or random according to the spatial statistics. Kappa values in bold differed significantly from zero (random classification) by z statistic (*p* < 0.05). The proportion of fields classified in the same spatial pattern of FWB is shown in parentheses. ^b^ Cohen’s Kappa paired agreement between tests. ^c^ Fleiss’s Kappa overall agreement among the tests.

## Data Availability

The data presented in this study are available on request from the corresponding author.

## Supplementary Materials

The following are available online at https://www.mdpi.com/article/10.3390/jof7080646/s1, Table S1: Description of fields assessed for Fusarium wilt of banana in Brazil, Table S2: Summary statistics used to study the progress of Fusarium wilt on bananas in six plots located in Teixeiras, Minas Gerais state, Brazil, from April 2017 to February 2019.
