# Supplementary material for: Incidence, Spatial Pattern and Temporal Progress of Fusarium Wilt of Bananas
Source: J Fungi (Basel). 2021 Aug 8;7(8):646. doi: 10.3390/jof7080646 (PMC8399182; doi:10.3390/jof7080646)
Supplement: Supplementary file 1 [file jof-07-00646-s001.zip › jof-1318730-supplementary.pdf]

Supplementary Materials for

Incidence, spatial pattern and temporal progress of Fusarium Wilt of bananas

Daniel Heck [1,#a], Miguel Dita [2], Emerson M. Del Ponte [1] and Eduardo S. G. Mizubuti [1,*]

[1] Universidade Federal de Viçosa, Viçosa, Minas Gerais, Brazil;
[2] Bioversity International, Cali, Colombia;
[#a] Current address: Cornell University, Geneva, New York, United States of America
[*] Correspondence: mizubuti@ufv.br;

This PDF file includes:

Table S1 to S2:

Table S1. Description of fields assessed for Fusarium wilt of banana in Brazil.

Field	Municipality, state [a]	Region [b]	Cultivar subgroup (genotype)	Area (ha)	Incidence ($\hat{p}$)
1	São Bento do Sapucaí, SP	SMZM	Pome (AAB)	2.82	1.66
2	São Bento do Sapucaí, SP	SMZM	Pome (AAB)	2.57	17.66
3	São Bento do Sapucaí, SP	SMZM	Pome (AAB)	2.08	4.82
4	São Bento do Sapucaí, SP	SMZM	Pome (AAB)	0.85	25.82
5	São Bento do Sapucaí, SP	SMZM	Pome (AAB)	1.29	7.92
6	Adolfo, SP	SJA	Silk (AAB)	6.67	41.42
7	Barbosa, SP	SJA	Silk (AAB)	3.86	4.94
8	Barbosa, SP	SJA	Silk (AAB)	3.33	36.9
9	Santa Mariana, PR	NPP	Silk (AAB)	6.40	11.49
10	Santa Mariana, PR	NPP	Silk (AAB)	2.23	32.77
11	Marinópolis, SP	SJA	Silk (AAB)	3.17	6.77
12	Marinópolis, SP	SJA	Silk (AAB)	2.78	12.39
13	Marinópolis, SP	SJA	Silk (AAB)	0.95	16.14
14	Palmeira d'Oeste, SP	SJA	Silk (AAB)	4.33	3.99
15	Marinópolis, SP	SJA	Silk (AAB)	0.99	19.42
16	Jacupiranga, SP	VR	Cavendish (AAA)	1.64	2.74
17	Jacupiranga, SP	VR	Pome (AAB)	2.43	10.42
18	Eldorado, SP	VR	Pome (AAB)	1.38	5.03
19	Jacupiranga, SP	VR	Pome (AAB)	1.35	3.26
20	Serra do Ramalho, BA	NMSF	Pome (AAB)	3.50	3.34
21	Janaúba, MG	NMSF	Pome (AAB)	5.94	0.09
22	Janaúba, MG	NMSF	Pome (AAB)	3.55	0.49

23	Jaíba, MG	NMSF	Pome (AAB)	2.34	4.44
24	Jaíba, MG	NMSF	Pome (AAB)	6.19	5.31
25	Corupá, SC	NC	Pome (AAB)	5.50	0.48
26	Corupá, SC	NC	Cavendish (AAA)	3.92	2.81
27	Corupá, SC	NC	Cavendish (AAA)	2.93	1.75
28	Corupá, SC	NC	Pome (AAB)	2.24	1.66
29	Corupá, SC	NC	Pome (AAB)	2.64	33.37
30	Teixeiras, MG	SMZM	Pome (AAB)	4.80	1.03

[a] SP: São Paulo; PR: Paraná; BA: Bahia; MG: Minas Gerais; SC: Santa Catarina.

[b] SMZM: Serra da Mantiqueira and Zona da Mata; SJA: São José do Rio Preto and Araçatuba; NPP: Norte Pioneiro Paranaense; VR: Vale do Ribeira; NMSF: Norte de Minas and Vale do São Francisco da Bahia; NC: Norte Catarinense.

Table S2. Summary statistics used to study the progress of Fusarium wilt on bananas in six plots located in Teixeiras, Minas Gerais state, Brazil, from April 2017 to February 2019.

Model	RMSE (± SD)	R^2 (± SD)	Best fit / *n*
Monomolecular	0.0049 ± 0.0007	0.969 ± 0.014	0 / 6
Logistic	0.0053 ± 0.0026	0.954 ± 0.048	1 / 6
Gompertz	0.0036 ± 0.0008	0.981 ± 0.012	5 /6

RMSE - Root mean square error; R^2 - Coefficient of determination; SD - Standard deviation.
